# Postoperative weight-bearing restrictions and rehabilitation after periacetabular osteotomy: a systematic review

**DOI:** 10.1186/s13018-025-06448-x

**Published:** 2025-10-29

**Authors:** Vincent J. Leopold, Alexander Hildebrandt, Esther Hübner, George Grammatopoulos, Paul E. Beaulé, Carsten Perka, Sebastian Hardt

**Affiliations:** 1 Center for Musculoskeletal Surgery, Charité – University Medicine Berlin, Charitéplatz 1, 10117 Berlin, Germany; 2 Charité – University Medicine Berlin, Berlin, Germany; 3 Division of Orthopaedic Surgery, University of Ottawa, The Ottawa Hospital, Ottawa, ON Canada

**Keywords:** Periacetabular osteotomy, Postoperative mobilization, Weight-bearing restrictions, Hip dysplasia, Rehabilitation

## Abstract

**Aims:**

This systematic review aimed to synthesize current evidence on postoperative rehabilitation strategies, particularly weight-bearing restrictions after periacetabular osteotomy (PAO).

**Methods:**

A systematic review was conducted following PRISMA 2020 guidelines. PubMed, Web of Science, and Embase were searched until January 12, 2025. After duplicate removal, studies were screened by title, abstract, and full text using predefined criteria. Studies were included if they reported postoperative weight-bearing protocols after isolated PAO; studies with additional procedures, non-human data, or lacking mobilization details were excluded. Primary endpoints included postoperative weight-bearing instructions, duration of partial weight-bearing, and brace use. Secondary endpoints included hip function, return to sports, and complications. Data extraction was performed independently by two reviewers. Bias was assessed using the MINORS tool.

**Results:**

The majority of studies recommended partial weight-bearing immediately postoperatively, commonly initiated on postoperative day one (18% of studies), typically lasting six (21%) to eight (18%) weeks. Variations included toe-touch, tip-touch, touch-down, flat-foot, protected, or restricted weight-bearing. Crutches were frequently recommended (48%). Bracing was infrequently reported (5%), with limited details provided. Return to sporting activities varied widely, typically recommended between six and twelve months postoperatively. Complication rates were diverse, with delayed weight-bearing showing a lower incidence of pelvic fractures compared to immediate full weight-bearing.

**Conclusion:**

This review highlights substantial variability and imprecise terminology in existing PAO mobilization protocols and a lack of standardization. Future research should prioritize prospective comparative studies to clarify safe, effective postoperative mobilization strategies. Establishing standardized, evidence-based rehabilitation guidelines could enhance patient outcomes, reduce complications, and decrease practice variability following PAO.

**Supplementary Information:**

The online version contains supplementary material available at 10.1186/s13018-025-06448-x.

## Introduction

Periacetabular osteotomy (PAO) is a well-established surgical procedure for the treatment of symptomatic hip dysplasia, aiming to improve hip stability and function while preventing the progression of osteoarthritis [1–4]. As a complex reconstructive procedure involving multiple osteotomies, the postoperative phase is critical to ensure adequate bone healing and optimize clinical outcomes [5, 6]. During the period of osseous consolidation, restrictions in weight-bearing are typically recommended to mitigate the risk of complications, such as loss of correction or implant failure [5, 7]. However, there is considerable heterogeneity in the protocols for postoperative mobilization and rehabilitation following PAO, with a lack of standardization [6]. Current clinical practices differ significantly in terms of the timing and intensity of weight-bearing, the duration of restricted loading, and the use of orthoses to support postoperative recovery [6]. These discrepancies may reflect an absence of high-quality evidence or consensus regarding optimal rehabilitation strategies. The primary aim of this systematic review was to identify and categorize postoperative mobilization strategies after PAO, with a focus on weight-bearing recommendations, the duration of partial or restricted loading, and the prescription of orthoses. Secondary endpoints included the assessment of hip function using validated patient-reported outcome measures (PROMs), the timeline for returning to sporting activities, and complication rates such as loss of correction, implant failure, and revision surgery.

By synthesizing the available evidence, this review aims to provide insights into existing postoperative rehabilitation protocols and highlight gaps in the literature to guide the development of evidence-based recommendations for clinical practice.

## Methods

### Search strategy

This systematic review was conducted according to the PRISMA 2020 guidelines. A comprehensive search was performed in PubMed, Web of Science, and OvidSP (including Embase). The search strategy combined MeSH and free-text terms: (‘periacetabular osteotomy’ OR ‘PAO’) AND (‘weight-bearing’ OR ‘mobilization’ OR ‘rehabilitation’ OR ‘postoperative care’. The search included studies published up to January 12, 2025.

### Study selection

We included randomized controlled trials, cohort studies, case–control studies, and case series of patients undergoing PAO without additional simultaneous procedures or irreversible mobilization restrictions. All levels of evidence were considered. Studies were excluded if they were not published in English, reported on additional concomitant surgeries, included patients with irreversible mobilization restrictions, or did not provide relevant data on postoperative mobilization or weight-bearing duration.

### Data extraction

Two reviewers independently extracted data on study characteristics, patient demographics, weight-bearing protocols, rehabilitation details, and outcomes using a standardized form. Extracted information included study-specific characteristics (first author, year of publication), postoperative weight-bearing restrictions, duration of partial weight-bearing, use of braces, total rehabilitation duration, hip function (e.g., Harris Hip Score, modified Harris Hip Score), participation in sports activities, follow-up duration and complication rate (major complications. Disagreements between reviewers were resolved through discussion or consultation with a third reviewer when necessary. The extracted data are summarized in Table 1.

**Table 1:** Overview of postoperative weight-bearing protocols following periacetabular osteotomy (PAO)

Study (first author and year)	Postoperative weight-bearing description	Duration of (partial) Weight-Bearing	Use of Braces	Total rehabilitation duration	Hip Function	Participation in sports activities	Follow-up duration	Complication rate (major)	MINORs Score
Baraka et al., 2022 [9]	pwb Full weight	6 w after	-	-	HHS: 70.8 + -4.9 →90.1 + -3.3	-	3.2y (2–5)	2/9 screw head irritation	18/24
Siebenrock et al., 2015 [10]	Mobi + Two crutches and pwb 15-20 kg	POD1 → 8w	-	-	-	-	11 y(9–12	4 revision surgery	9/16
Löchel et al., 2021[11]	Immediate pwb 15 kg, Full weight	4 w after	-	-	-	-	-	No major complications	11/16
O’Connor et al., 2024 [12]	Early mobi Few steps stairs	POD1 1;2 days 2.5;4 days	-	-	-	-	-	-	7/16
Ahmad et al., 2024 [13]	surgeon-led Mobi + crutches Pwb 15 kg	POD1 4 w	-	-	-	-	-	-	15/24
Leunig et al., 1998 [14]	Mobi Pwb 5-10 kg, Full weight	POD2 After 8 w	-	-	-	-	-	-	7/16
Albertz et al., 2021 [15]	Mobi Toe-touch-wb, Sit, stand[1],stairs[4] with crutches	POD1	-	-	-	-	-	-	19/24
Peters et al., 2015 [16]	Walk 0(0–15) 20(0–75) 77.5(25–150) Feet	POD1 POD2 POD3	-	-	-	-	-	-	19/24
Leunig et al., 2017 [17]	Toe-touch-wb	POD1	-	-	-	-	-	-	8/16
Leopold et al., 2021 [7]	Tip-touch-wb; Half of body-weight; Increase to full weight	6 w; 7th-10th 10th-3 months	-	-	-	-	82.04(67–101)d; 81.53(70–103)d	Implant removal: 34/93 36.6%	13/24
Kamath et al., 2016 [18]	Pwb 15 kg Full weight	4–6 w At 8 w	-	-	-	Stationary bike at 4 w; Return 6–12 m	-	-	4/16
Ito et al., 2014 [19]	1. Mobi; Pwb crutches; Full two crutches 2. Mobi; full weight + crutches	POD1 2–4 w 8w-12w POD0 POD1-8w	-	Walking without support 1.6.9 m(2.5–15) 2. 4.2 m(2–10.5)	HHS: 1:68.5 + -8.4 →91.6 + -8.8 2: 68.9 + -8.5 →89.8 + -9.3	-	2 y	More pelvic fractures in 2 1/80 vs. 8/76	8/16
Kinoshita et al., 2024 [20]	1.pwb 10 kg 2.pwb 20 kg Both: crutches Wb increase 10 kg	POD 2/3 At 2–4 w Every 2w	-	-	-	-	1y	Delayed union: 3.5% (late pwb); 22% (early pwb)	15/24
Yoshimoto et al., 2020 [21]	Pwb + Crutches Wb gradually increased Full weight	1/2 w At 5–8 w	-	-	-	-	1y	-	16/24
Naito et al., 2014 [22]	Pwb 10 kg crutches Full weight	POD3 8 w	-	-	HHS: 78.08→95.36	-	1y	0	5/16
Sucato et al., 2010 [23]	Pwb 20–30 pounds Full + crutches Full weight	6 w 12 w	-	-	mHHS (max 89): 64.6→74.5	-	1y	-	12/16
Dienst et al., 2018 [24]	Mobi bedside Walking to toilet Pwb 20 kg + crutches increase by 10 kg with physio to full weight	POD1 POD2 For 6 w Xray controll 10-12w	-	-	mHHS: 87.6 + -13.9	-	20.4 + -10.3 m	0	8/16
Jacobsen et al., 2014 [25]	Pwb max. 30 kg Full weight	First 6-8w After	-	-	-	-	1y	-	12/16
Takahashi et al., 2020 [26]	One-third pwb Full weight	At 21d At 14w	-	-	-	Return 12.7 + -10.8 m	34.1 m + -17.2	-	10/16
Disantis et al., 2022 [6]	Foot-flat-wb 25% + crutches Wb progression gradually	For 6–8 w At 6-12w	-	-	-	Stationary bike at 6-8w Return: 26 + w	-	-	8/16
Mechlenburg et al., 2007 [27]	Mobi Pwb 30 kg + crutches Full weight	POD2 Until 8w After	-	-	-	-	6 m	0	12/16
Hamai et al., 2014 [28]	Pwb + crutches Gradual increase to full weight	1 and 2 w At 5-8w	-	-	-	-	46.1 m(12–120)	4.7% ischio-pubic fractures	8/16
Fujita et al., 2022 [29]	Mobi 1.Pwb 10 kg Increase 10 kg 2. Pwb 20 kg Increase 10 kg	POD1 1.POD2 Every 2w At 2w Every 2w	-	-	-	-	12,4 m(12–16)	Delayed union 6 patients	10/16
Evans et al., [30]	Discharged Physio, progressive wb	POD2 At 4w-12w	-	-	-	-	2.5y	-	8/16
Gu et al., 2021 [31]	Pwb Increased Full weight	Within 6w After 6w 12w	-	-	mHHS: 70→91	-	18 m (12–27)	0	10/16
Klahs et al., 2021 [32]	Toe-touch-wb Full weight	For 6w 12w	-	-	-	Full activity at 6 m 1. volleyball 12 m	2y	-	8/16
Kaneuji et al., 2021 [33]	1/3 wb Full weight	At 3w 8w	-	-	HHS: 57.9 (25–83)→89.6 (62–100)	-	2y	0	10/16
Seo et al., 2018 [34]	Active motion Pwb 10 kg + 2 crutches Full weight	POD1 POD3 At 8w	-	-	-	-	4.8y (2–7.2)	-	10/16
Arpey et al., 2018 [35]	Flat-foot-touch-wb	For 12w	Brace was worn post-op	-	-	-	1y	-	8/16
Maranho et al., 2018 [36]	Wheelchair non-wb Protected wb (walker)	For 4-6w Additional 4-6w	-	-	HHS post: 91 (65–96)	-	13.1 + -5.2y	8% major	12/16
Sheean et al., 2017 [37]	Protected wb	For 8w	-	-	mHHS: 41,8→ 100	-	15 m	0	8/16
Sankar et al., 2017 [38]	Pwb Progressive wb	4-6w after	-	-	mHHS: 62→	-	-	-	14/16
Novais et al., 2016 [39]	Pwb 20–30% + crutches Full weight	First 8-12w after	-	-	mHHS: 63→88; 71→86	-	5.2y (2–16)	33%; 13% grade 2 or higher complication	18/24
Collado et al., 2016 [40]	Toe-touch-wb Full weight	For 6w At 8w	-	-	HHS: 39→86	-	3y	-	8/16
Luo et al., 2015 [41]	Mobi with crutches Pwb Full weight	POD1 For 6-8w 10-12w	-	-	HHS: 96,100→	-	3 m	-	8/16
Swarup et al., 2015 [42]	Early mobi Pwb Full weight	First 6w After 6w	-	-	-	-	-	-	4/16
Hingsammer et al., 2015 [43]	Mobi with crutches Pwb 1/6 body weight Full weight	After surgery For 4w after	-	-	-	Return to full activity by 4-6 m	2y	-	12/16
Nassif et al., 2012 [44]	Pwb 30 lb Progressive wb Full weight	For 6w After At 16w	-	-	mHHS: 64.3 + -13.2→ 87.4 + -14.2	-	3.4y (2–9.7)	6/88 (1 delayed union)	18/24
Ito et al., 2011 [45]	Non-wb Pwb + crutches 1 crutch	First 2w At 2-4w For 12w	-	-	HHS: 70→90	-	11y (5–20)	-	18/24
Yamanaka et al., 2011 [46]	Walk + crutches Full weight	After 1w After 8w	-	-	HHS: 85 →96	Return to full activity (skiing) at 4 m	2y	-	8/16
Teratani et al., 2011 [47]	Active motion Pwb + crutches Full weight	POD2 POD3 At 8w	-	-	HHS: 69.6→ 90.9; 71.1→ 91.8	-	2y	-	18/24
Matheney et al., 2010 [48]	Pwb Progressed to full weight	POD2/3 By 6-8w	-	-	-	-	9y	20/ 109	12/16
Thawrani et al., 2010 [49]	Pwb 9–13,6 kg Full weight	For 6w after	-	-	-	-	2y	3/76 major (osteonecrosis femoral head)	12/16
Troelsen et al., 2009 [50]	Pwb + crutches	First 8w	-	-	-	-	6.8y	-	12/16
Keogh et al., 2008 [51]	Toe-touch-wb	6-8w	-	-	-	Return from 25w	-	-	n/a
Peters et al., 2006 [52]	Pwb + crutches Full + 1 crutch Walking w/o limp	For 6w For 6w At 12w	-	-	HHS: 54→87	-	46 m	10 major complications (nerve palsies)	12/16
Clohisy et al., 2005 [53]	pwb	First 8w	-	-	HHS: 73.4→ 91.3	-	4.2y	2/13 (non-union; loss of fixation)	12/16
Ganz et al., 2004 [54]	Mobi + pwb 10 kg + crutches Walk with cane	POD3 After 8-10w	Soft splint	-	-	-	-	14 major (1 non-union; 13 implant removal)	10/16
Hsieh et al., 2003 [55]	Pwb + crutches Walk with cane w/o cane	POD4/5 After 6w At 12w	-	-	Merle d’Aubigne and Postel hip score: 13.2→17	-	4.2y	0	10/16
Ko et al., 2002 [56]	Mobi Pwb Full weight	POD4/5/6 At 1w At 12-16w	-	-	mHHS: 59.1 + -15.8 → 87.97 + -14.3	-	5.5y	-	10/16
Crowther et al., 2002 [57]	Touch-down-wb	12w	Abduction brace 12w	-	-	-	2y	-	8/16
Xiang et al., 2022 [58]	Active/passive exercise Toe-touch-wb Full weight	POD1 First 12w after	-	-	-	-	12 m (12–36)	No major	10/16
Swarup et al., 2021 [59]	20% pwb Wb as tolerated + crutches	For 4w Until 6w	-	-	mHHS: 50→ 88	Return to all activities at 6 m	Min. 1y	0	10/16
Matsuda et al., 2016 [60]	Discharged on Pwb + crutches Full weight	POD3 At 6w	-	-	-	-	-	0	8/16
Buchler et al., 2014 [61]	Passive motion Restricted wb 15 kg Increased wb	POD1 For 8w after	-	-	-	-	-	-	n/a
Karashima et al., 2011 [62]	Pwb 10 kg + crutches Full weight	POD3 After 8w	-	-	HHS: 1. 73.9→ 94.3; 2. 76.7→ 94.7	-	70.9 m; 70.6 m	12/ 191 (2 pubic non-union; 7 pubic fracture; 1 ischial fracture)	18/24
Stetzelberger et al., 2021 [63]	Pwb 15 kg Increase wb	For 8w after	-	-	-	-	22 + -6y	-	14/16
Polkowski et al., 2014 [64]	Mobi Toe-touch-wb	POD2 POD3	-	-	-	-	26 m (1–96)	-	12/16
Albers et al., 2013 [65]	Mobi + crutches Pwb 15 kg Full weight	Early For 8w after	-	Rehab: 2-3 m	Merle d’Aubigné: 15→16; 15→16	-	11y (10–14)	-	20/24
Mayman et al., 2002 [66]	Touchdown- wb + crutches Progressive wb	For 6w after	-	-	-	-	-	0	10/16
Salih et al., 2020 [67]	Pwb 20 kg + crutches Progressed to 30 kg Full weight + crutches w/o crutches	For 6w For 3w After By 12w	-	-	-	Impact exercise at 5-6 m	26 m	2.7% major ( 1 revision fixation; 2 stress fractures)	10/16
Leopold et al., 2023 [68]	Tip-touch-pwb Increase to half body-weight Increase to full weight	First 6w Till 10th w 10- 12w	-	-	Subjective hip value 41.9→77.9; 42.4→82.4	-	63 m + -10	3/120 (implant migration)	17/24
Leopold et al., 2021 [63]	Tip-touch-pwb Increased half body-weight Increase to full weight	First 6w 7th-10th w 10- 12w	-	-	-	-	94d (70–112)	No major	14/24

This table summarizes the different studies evaluating weight-bearing protocols after PAO, including the duration of partial and full weight-bearing, the use of braces, total rehabilitation time, functional outcomes, participation in sports, follow-up duration, and major complications. Abbreviations: pwb – Partial weight-bearing; wb – Weight-bearing; POD – Postoperative day; HHS – Harris Hip Score; mHHS – Modified Harris Hip Score

### Data synthesis

A narrative synthesis was performed due to heterogeneity in study designs, rehabilitation protocols, and outcome measures among the included studies. Findings were systematically categorized based on postoperative weight-bearing strategies, mobilization timelines, and clinical outcomes related to function and mobility. Results were reported following the Preferred Reporting Items for Systematic Reviews and Meta-Analyses (PRISMA) guidelines, and a PRISMA-compliant flowchart illustrating the detailed study selection process was provided.

### Risk of bias and quality assessment

The risk of bias and methodological quality of non-randomized studies were assessed using the Methodological Index for Non-Randomized Studies (MINORS) tool. This allowed for an objective evaluation of study design, risk of bias, and internal validity. The MINORS scoring system comprises 12 criteria for comparative studies and 8 criteria for non-comparative studies, each scored on a scale from 0 to 2 (0 = not reported, 1 = reported but inadequate, 2 = reported and adequate), resulting in maximum scores of 24 and 16 points respectively. Comparative studies were further assessed for baseline comparability, statistical methodology, and control of confounders.

## Results

**Fig. 1:**
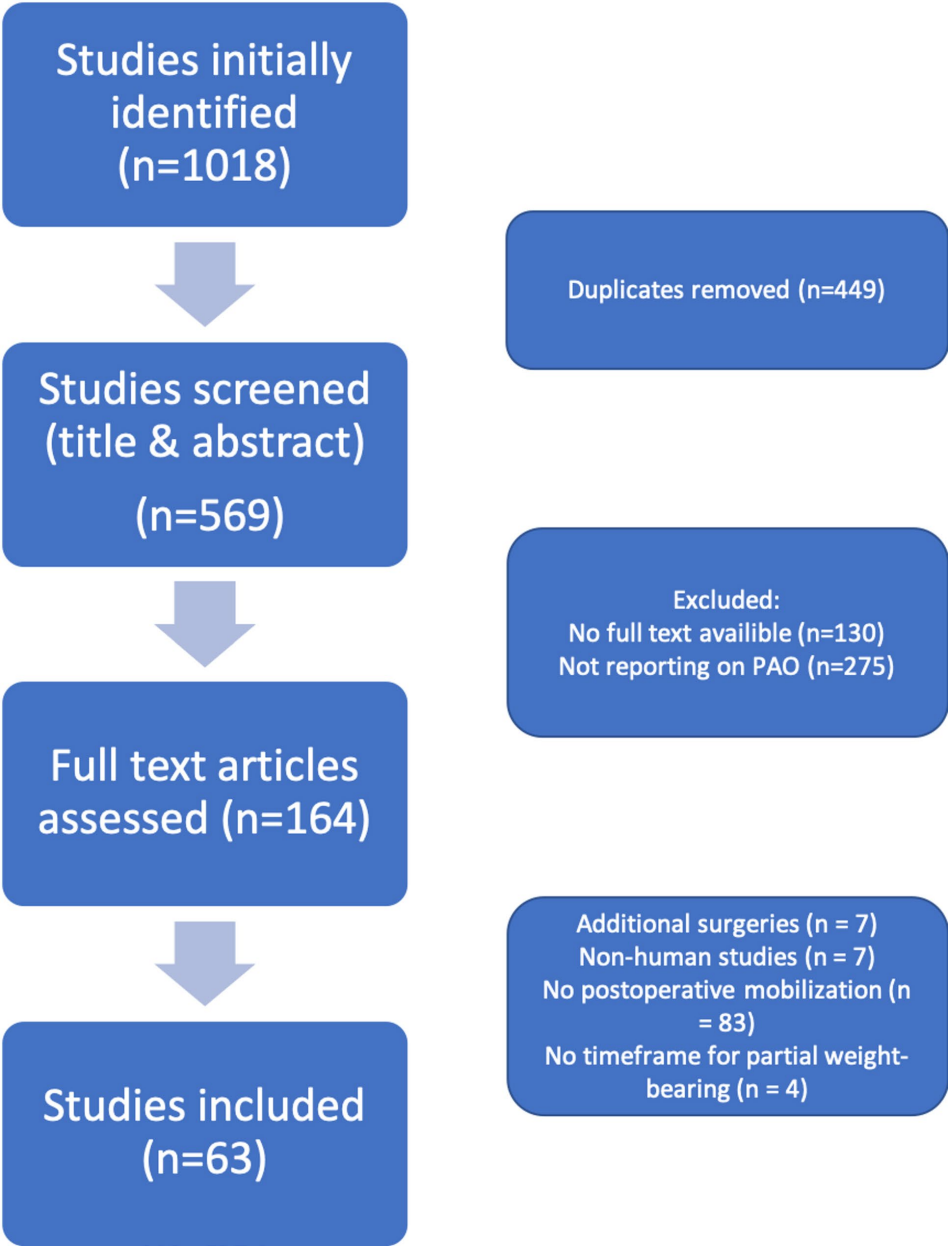
Flowchart of study inclusion illustrating the study selection process for this systematic review

The selection process followed the PRISMA flow diagram. A total of 1018 studies were initially identified. After removing 449 duplicates, 569 studies were screened based on title and abstract. Of these, 130 studies were excluded due to lack of full-text availability, and 275 studies did not report on periacetabular osteotomy. This resulted in 164 full-text articles assessed for eligibility based on predefined inclusion and exclusion criteria. Among these, 7 studies involved patients undergoing additional surgeries, 7 studies were not conducted on humans, 83 studies did not report on postoperative mobilization, and 4 studies did not specify a time frame for partial weight-bearing. Ultimately, 63 studies were included in the final analysis [5, 6, 8–68]. For a detailed illustration of study study inclusion see Fig. 1.

### Primary endpoints

#### Weight-bearing descriptions

A total of 18 studies (29%) reported the initiation of postoperative mobilization. The majority (11 studies, 18%) described mobilization starting on postoperative day one. Additional studies reported mobilization on postoperative day two (4 studies, 6%), day three (1 study, 2%), day four (1 study, 2%), or on the day of surgery (1 study, 2%).

Partial weight-bearing was the most frequently reported postoperative strategy, mentioned in 41 studies (65%). Specific weight-bearing descriptions varied: 7 studies (11%) used toe-touch weight-bearing, 3 studies (5%) used tip-touch weight-bearing, 2 studies (3%) used touch-down weight-bearing, 2 studies (3%) used flat-foot weight-bearing, 2 studies (3%) used protected weight-bearing, and 1 study (2%) used restricted weight-bearing. The use of crutches as an assistive device was reported in 30 studies (48%).

#### Duration of partial weight-bearing

The prescribed duration of partial weight-bearing was reported in 58 studies (85%). Among these, the most common duration was six weeks, recommended in 14 studies (21%), followed by eight weeks in 12 studies (18%). A duration of six to eight weeks was noted in 6 studies (9%), and four to six weeks in 4 studies (6%). Durations of 10 to 12 weeks were recommended in 3 studies (5%), while 12 weeks and two to four weeks were each reported in 4 and 3 studies, respectively. Less frequent regimens included five to eight weeks (2 studies, 3%), eight to ten weeks (1 study, 2%), eight to twelve weeks (1 study, 2%), twelve to sixteen weeks (1 study, 2%), and four to twelve weeks (1 study, 2%).

For a detailed overview of partial weight-bearing duration and progression to partial weight bearing see Table 1.

#### Use of braces

Braces were mentioned in 3 studies (5%). However, none of these studies provided details regarding the specific type of brace used.

Table 1 summarizes the different studies evaluating weight-bearing protocols after PAO, including the duration of partial and full weight-bearing, the use of braces, total rehabilitation time, functional outcomes, participation in sports, follow-up duration, and major complications. Abbreviations: pwb–Partial weight-bearing; wb–Weight-bearing; POD–Postoperative day; HHS–Harris Hip Score; mHHS–Modified Harris Hip Score.

### Secondary endpoints

#### Hip function outcomes

A total of 25 studies (40%) reported hip function outcomes using four different scoring systems. The median Harris Hip Score (HHS) improved from 70 to 90 in 13 studies (21%). The modified Harris Hip Score (mHHS) showed an improvement from 60 to 90 in 9 studies (14%). The Merle d’Aubigné score increased from 14 to 16.5 in 2 studies (3%). The Subjective Hip Value (SHV) improved from 40 to 80 in 1 study (2%).

#### Return to sports

Return to sports activities was reported in 9 studies (14%). The most common recommendation was a return after six months, documented in 4 studies (6%). Two studies (3%) suggested a return timeframe of six to twelve months, depending on the intensity of activity. One study each (2%) recommended return after four months, four to six months, or twelve months.

#### Complication rates

Complications were described in 31 studies (49%). Among these, 10 studies (16%) explicitly reported no complications, while 3 studies (5%) noted no major complications. Specific minor complications included implant removals (36.6% in one study), delayed union (22% in early weight-bearing vs. 3.5% in delayed weight-bearing in one study), and pelvic fractures (10% in one study with early full weight-bearing). Additional complications included nerve palsies, stress fractures, osteonecrosis, and loss of fixation. For a detailed overview see Table 1.

## Discussion

This systematic review highlights significant variability in postoperative rehabilitation strategies following PAO. The findings indicate that while partial weight-bearing is commonly recommended, the duration and progression to full weight-bearing vary considerably. Most studies prescribed partial weight-bearing for six to eight weeks, with full weight-bearing typically allowed between eight to twelve weeks. However, there is still no consensus on the optimal timing of weight-bearing. Additionally, postoperative bracing was infrequently used, and reporting on postoperative mobilization strategies was inconsistent [35]. Functional outcomes demonstrated significant improvements in hip scores, while the return to sports was highly variable, often recommended after six to twelve months [5, 8, 10, 22–24, 31, 33, 36, 37, 39, 41, 44–47, 52, 53, 55, 62, 65, 68]. Complication rates varied across studies, with early full weight-bearing associated with an increased risk of fractures and implant-related complications. These findings underscore the need for standardized, evidence-based rehabilitation protocols to optimize recovery and minimize complications.

Our systematic review revealed considerable variation and imprecise terminology in the reporting of weight-bearing protocols following periacetabular osteotomy. This inconsistency is not unique to PAO literature; similar issues have been reported in orthopedic studies as highlighted by Trompeter et al., who systematically reviewed weight-bearing instructions in studies of musculoskeletal trauma [69]. The authors identified substantial heterogeneity and ambiguity in terminology, prompting a consensus recommendation to standardize definitions and improve clarity in future research [69]. Their conclusion emphasized the need for clearly defined, universally accepted terms to ensure reproducibility and clinical applicability [69]. The findings of our review reinforce this call for standardization, underscoring the necessity for consistent descriptions of postoperative mobilization and weight-bearing in PAO studies to facilitate comparison and translation into clinical practice.

A recent Delphi consensus by Disantis et al. (2022) provides further insight into the importance of standardizing postoperative rehabilitation guidelines [6]. The expert panel, consisting primarily of physiotherapists, reached consensus on early postoperative weight-bearing precautions, recommending 25% foot-flat weight-bearing for six to eight weeks, aligning with the majority of studies in this review. However, it must be emphasized that this timeline represents expert opinion rather than an evidence-based recommendation. Furthermore, the guidelines emphasize a structured progression of rehabilitation exercises, highlighting the need for gradual strengthening and neuromuscular control to optimize recovery and minimize complications [6]. Importantly, surgical factors, including fixation type, osteotomy stability, and correction magnitude, must also be evaluated by the surgeon when determining postoperative restrictions. The standardization proposed by Disantis et al. may thus serve as a foundation for improving consistency in postoperative protocols and patient outcomes [6]. Notably, the authors state that no studies currently exist supporting specific weight-bearing and range of motion (ROM) precautions, therapeutic exercise prescription, or metrics for clearance to return-run and return-to-sport. Therefore, the Delphi method was utilized to generate expert opinion in a content area where evidence is lacking [6].

Although clear evidence is limited, a study by Ito et al. (2014) demonstrated that immediate full weight-bearing following PAO was associated with a significantly higher incidence of postoperative pelvic fractures compared to a delayed weight-bearing approach [5]. In their study, one group was allowed to fully weight-bear immediately after surgery, while the other group delayed full weight-bearing until two months postoperatively. The results showed a significantly higher fracture rate in the immediate full weight-bearing group (10.5% of cases) compared to the delayed group (1.25%). These findings suggest that initial partial weight-bearing is imperative for early bone consolidation and the structural integrity of the pelvis [5].

Biomechanical evidence further supports these findings. A study by Kaku et al. demonstrated that following PAO, altered load transmission patterns lead to increased stress on the inferior pubic ramus, ischium, and posterior column [70]. Their finite element analysis revealed that coronal pelvic inclination significantly increases tensile stress on these bony structures, thereby predisposing patients to stress fractures. These findings highlight the necessity of controlled postoperative weight-bearing progression to allow adequate bony consolidation and healing, further justifying recommendations for an initial period of restricted weight-bearing [70].

Apart from the study by Ito et al., which compared two extreme opposites of postoperative rehabilitation and weight-bearing restrictions after PAO, comparative studies evaluating different durations of partial weight-bearing following PAO are lacking in the literature [5]. This systematic review has demonstrated significant variability in the reported durations of partial weight-bearing and the transition to full weight-bearing. Future comparative studies should aim to determine how quickly weight-bearing can be increased after PAO without compromising patient safety.

A shorter period of postoperative partial weight-bearing is desirable and beneficial, provided it is safe. Prolonged unloading after PAO can lead to muscle atrophy, neuromuscular deficits, joint stiffness, and an increased risk of thromboembolic events, which may delay functional recovery and prolong rehabilitation [64, 71–74]. Early weight-bearing, when introduced cautiously, helps preserve muscle strength, improves circulation, and reduces these risks while supporting a faster return to daily activities, work, and sports.

Beyond its effects on functional recovery, early controlled weight-bearing may also enhance bone healing. Mechanical loading has been shown to stimulate osteoblast activity and promote bone remodeling, which is essential for bony consolidation of osteotomy sites [75, 76].

These findings underscore the comprehensive benefits of early mobilization, including reduced neuromuscular deficits, reduced muscle atrophy, faster functional recovery, and enhanced bone healing.

However, the timing of full weight-bearing must strike a balance between biological healing and functional demands — a "race" between fracture healing and the return to function. Optimal outcomes depend on achieving sufficient stability for bone healing while facilitating early mobilization. This balance is influenced by several factors. Patient-related factors include age, sex, bone mineral density, smoking status, metabolic health, and adherence to postoperative instructions. Surgical factors include the type and rigidity of fixation, the magnitude of acetabular correction, and the specific osteotomy technique [77, 78]. Future studies should therefore aim to define patient-specific and surgery-specific thresholds for safe weight-bearing progression to optimize both safety and functional recovery.

This review has several limitations. The included studies exhibit considerable heterogeneity in methodology, patient populations, and rehabilitation protocols, limiting direct comparability. Additionally, the lack of randomized controlled trials reduces the overall quality of evidence. Variability in the definitions of postoperative mobilization and weight-bearing further complicates data interpretation. Moreover, differences in outcome assessment tools and follow-up durations across studies hinder a consistent evaluation of long-term results. Finally, no prospective comparative studies directly evaluating different mobilization strategies were available, emphasizing the need for future research to establish standardized, evidence-based rehabilitation protocols.

## Conclusion

This systematic review highlights the considerable variability in postoperative mobilization strategies following periacetabular osteotomy (PAO). While partial weight-bearing is commonly recommended, the duration and progression to full weight-bearing vary considerably. The lack of comparative studies evaluating different weight-bearing timelines limits the ability to establish evidence-based guidelines.

Future research should focus on optimizing postoperative mobilization protocols by identifying safe and effective weight-bearing progression strategies. Prospective comparative studies are needed to determine the ideal timeline for increasing weight-bearing after PAO while balancing safety and functional recovery. Standardized rehabilitation guidelines based on high-quality evidence could improve patient outcomes and reduce practice variability in postoperative care.

## Supplementary Information

Below is the link to the electronic supplementary material.

Supplementary Material 1

Supplementary Material 2

## Data Availability

The data underlying this article will be shared upon reasonable request from the corresponding author.
