# Supplementary material for: Postoperative weight-bearing restrictions and rehabilitation after periacetabular osteotomy: a systematic review
Source: J Orthop Surg Res. 2025 Oct 29;20:944. doi: 10.1186/s13018-025-06448-x (PMC12573821; doi:10.1186/s13018-025-06448-x)
Supplement: Supplementary file 1 — Supplementary Material 1 [file 13018_2025_6448_MOESM1_ESM.docx]

| Study (First Author and Year) | Level of evidence | Number Cases (% female) | Mean age | Postoperative Weight-Bearing description | Duration of (partial) Weight-Bearing | Use of Braces | Total Rehabilitation Duration | Hip Function | Participation in Sports Activities | Follow-Up Duration | Complication Rate (major) | MINORs Score |
| --- | --- | --- | --- | --- | --- | --- | --- | --- | --- | --- | --- | --- |
| Baraka et al., 2022 (10) | IV | 9 (78%) | 22.4 years (range 15–30) | pwb  Full weight | 6 w  after | - | - | HHS:  70.8 +-4.9 🡪90.1+-3.3 | - | 3.2y (2-5) | 2/9 screw head irritation | 18/24 |
| Siebenrock et al., 2015 (11) | IV | 29 (62%) | 27 years (range 14–46) | Mobi+Two crutches and pwb 15-20kg | POD1  🡪  8w | - | - | - | - | 11 y(9-12 | 4 revision surgery | 9/16 |
| Löchel et al., 2021(12) | III | 217 (79.5%) | 29 years (14–50) | Immediate pwb 15kg,  Full weight | 4 w  after | - | - | - | - | - | No major complications | 11/16 |
| O'Connor et al., 2024 (13) | II | 230 patients (84%) | 26.7 years (range 14–52) | Early mobi  Few steps  stairs | POD1  1;2days  2.5;4days | - | - | - | - | - | - | 7/16 |
| Ahmad et al., 2024 (14) | III | 150 (82.7%) | 27.8 years (range 15–45) | surgeon-led Mobi +crutches  Pwb 15kg | POD1   4 w | - | - | - | - | - | - | 15/24 |
| Leunig et al., 1998 (15) | IV | 75 (85%) | 28 years (range 15–48) | Mobi  Pwb 5-10kg,  Full weight | POD2  After 8 w | - | - | - | - | - | - | 7/16 |
| Albertz et al., 2021 (16) | III | 32 (90.6%) | 15.9 years (range 13–21) | Mobi  Toe-touch-wb,  Sit, stand(1) ,stairs(4) with crutches | POD1 | - | - | - | - | - | - | 19/24 |
| Peters et al., 2015 (17) | III | 75 (87%) | 22 years (13–43) | Walk  0(0-15)  20(0-75)  77.5(25-150)  Feet | POD1  POD2  POD3 | - | - | - | - | - | - | 19/24 |
| Leunig et al., 2017 (18) | IV | 1 (100%) | 12 years | Toe-touch-wb | POD1 | - | - | - | - | - | - | 8/16 |
| Leopold et al., 2021 (8) | III | 84 patients (79%) | 27 years (range 15–45; group 1 = 26.5, group 2 = 28.4) | Tip-touch-wb;  Half of body-weight;  Increase to full weight | 6 w;  7^th^-10^th^  10^th^-3 months | - | - | - | - | 82.04(67-101)d; 81.53(70-103)d | Implant removal: 34/93 36.6% | 13/24 |
| Kamath et al., 2016 (19) | V | NA | NA | Pwb 15kg  Full weight | 4-6 w  At 8 w | - | - | - | Stationary bike at 4 w;  Return 6-12 m | - | - | 4/16 |
| Ito et al., 2014 (5) | III | 154 (90%) | 32 years (range 13–52) | 1. Mobi;  Pwb crutches;  Full two crutches  2. Mobi;  full weight+ crutches | POD1  2-4 w  8w-12w  POD0  POD1-8w | - | Walking without support  1.6.9m(2.5-15)  2. 4.2m(2-10.5) | HHS:  1:68.5+-8.4 🡪91.6+-8.8  2: 68.9+-8.5 🡪89.8+-9.3 | - | 2 y | More pelvic fractures in 2.  1/80 vs. 8/76. | 8/16 |
| Kinoshita et al., 2024 (20) | III | 86 (90%) | 32 years (range 16–51) | 1.pwb 10kg  2.pwb 20kg  Both: crutches  Wb increase 10kg | POD 2/3  At 2-4 w  Every 2w | - | - | - | - | 1y | Delayed union: 3.5% (late pwb); 22% (early pwb) | 15/24 |
| Yoshimoto et al., 2020 (21) | II | 14 (86%) | 24 years (16–38) | Pwb +Crutches  Wb gradually increased  Full weight | 1/2 w  At 5-8 w | - | - | - | - | 1y | - | 16/24 |
| Naito et al., 2014 (22) | IV | 22 (86%) | 32 years (15–52) | Pwb 10kg crutches  Full weight | POD3  8 w | - | - | HHS: 78.08🡪95.36 | - | 1y | 0 | 5/16 |
| Sucato et al., 2010 (23) | IV | 21 (71%) | 16.1 years (12–19) | Pwb 20-30 pounds  Full+crutches  Full weight | 6 w  12 w | - | - | mHHS (max 89): 64.6🡪74.5 | - | 1y | - | 12/16 |
| Dienst et al., 2018 (24) | IV | 34 (76%) | 28 years (15–46) | Mobi bedside  Walking to toilet  Pwb 20kg+ crutches  increase by 10 kg with physio to full weight | POD1  POD2  For 6 w  Xray controll  10-12w | - | - | mHHS: 87.6+-13.9 | - | 20.4+-10.3 m | 0 | 8/16 |
| Jacobsen et al., 2014 (25) | II | 23 (83%) | 32 years (18–53) | Pwb max. 30kg  Full weight | First 6-8w  After | - | - | - | - | 1y | - | 12/16 |
| Takahashi et al., 2020 (26) | II | 43 (91%) | 32 years (18–55) | One-third pwb  Full weight | At 21d  At 14w | - | - | - | Return 12.7+-10.8m | 34.1m+-17.2 | - | 10/16 |
| Disantis et al., 2022 (6) | NA | NA | NA | Foot-flat-wb 25%+ crutches  Wb progression gradually | For 6-8 w  At 6-12w | - | - | - | Stationary bike at 6-8w  Return: 26+ w | - | - | 8/16 |
| Mechlenburg et al., 2007 (27) | IV | 32 (84%) | 39 years (20–57) | Mobi  Pwb 30kg+ crutches  Full weight | POD2  Until 8w  After | - | - | - | - | 6m | 0 | 12/16 |
| Hamai et al., 2014 (28) | IV | 275 (89%) | 42.4 years (13–64) | Pwb+ crutches  Gradual increase to full weight | 1 and 2 w  At 5-8w | - | - | - | - | 46.1m(12-120) | 4.7% ischio-pubic fractures | 8/16 |
| Fujita et al., 2022 (29) | IV | 83 (90%) | 37 years (range 18–55) | Mobi  1.Pwb 10kg  Increase 10kg  2. Pwb 20kg  Increase 10 kg | POD1  1.POD2  Every 2w  At 2w  Every 2w | - | - | - | - | 12,4m(12-16) | Delayed union 6 patients | 10/16 |
| Evans et al., (30) | IV | 2 (NA) | NA | Discharged  Physio, progressive wb | POD2  At 4w-12w | - | - | - | - | 2.5y | - | 8/16 |
| Gu et al., 2021 (31) | III | 44 (91%) | 31.2 years (SD ±9.4) | Pwb  Increased  Full weight | Within 6w  After 6w  12w | - | - | mHHS: 70🡪91 | - | 18m (12-27) | 0 | 10/16 |
| Klahs et al., 2021 (32) | IV | 1 (100%) | 14 years | Toe-touch-wb  Full weight | For 6w  12w | - | - | - | Full activity at 6m  1. volleyball 12m | 2y | - | 8/16 |
| Kaneuji et al., 2021 (33) | IV | n = 52 (88%) | 38 years (17–56) | 1/3 wb  Full weight | At 3w  8w | - | - | HHS: 57.9 (25-83)🡪89.6 (62-100) | - | 2y | 0 | 10/16 |
| Seo et al., 2018 (34) | IV | 46 patients (83%) | 32 years (13–55) | Active motion  Pwb 10kg+2 crutches  Full weight | POD1  POD3  At 8w | - | - | - | - | 4.8y (2-7.2) | - | 10/16 |
| Arpey et al., 2018 (35) | IV | 1 (female) | 17 years | Flat-foot-touch-wb | For 12w | Brace was worn post-op | - | - | - | 1y | - | 8/16 |
| Maranho et al., 2018 (36) | III | 39 (85%) | 15.5 years (12–19) | Wheelchair non-wb  Protected wb (walker) | For 4-6w  Additional 4-6w | - | - | HHS post: 91 (65-96) | - | 13.1+-5.2y | 8% major | 12/16 |
| Sheean et al., 2017 (37) | IV | 16 (81%) | 15 years (12–18) | Protected wb | For 8w | - | - | mHHS: 41,8🡪 100 | - | 15m | 0 | 8/16 |
| Sankar et al., 2017 (38) | III | 391 (89%) | 25 years (13–45) | Pwb  Progressive wb | 4-6w  after | - | - | mHHS: 62🡪 | - | - | - | 14/16 |
| Novais et al., 2016 (39) | III | 56 (89%) | 16 years (13–21) | Pwb 20-30% + crutches  Full weight | First 8-12w  after | - | - | mHHS: 63🡪88; 71🡪86 | - | 5.2y (2-16) | 33%; 13% grade 2 or higher complication | 18/24 |
| Collado et al., 2016 (40) | IV | 1 (0%) | 20 years | Toe-touch-wb  Full weight | For 6w  At 8w | - | - | HHS: 39🡪86 | - | 3y | - | 8/16 |
| Luo et al., 2015 (41) | III | 56 (91%) | 27 years (18–41) | Mobi with crutches  Pwb  Full weight | POD1    For 6-8w  10-12w | - | - | HHS: 96🡪100 | - | 3m | - | 8/16 |
| Swarup et al., 2015 (42) | NA | NA | NA | Early mobi  Pwb  Full weight | First 6w  After 6w | - | - | - | - | - | - | 4/16 |
| Hingsammer et al., 2015 (43) | II | 26 (85%) | 25 years (13–43) | Mobi with crutches  Pwb 1/6 body weight  Full weight | After surgery  For 4w  after | - | - | - | Return to full activity by 4-6m | 2y | - | 12/16 |
| Nassif et al., 2012 (44) | III | 88 (74%) | 19 years (13–27) | Pwb 30lb  Progressive wb  Full weight | For 6w  After  At 16w | - | - | mHHS: 64.3 +-13.2🡪 87.4 +-14.2 | - | 3.4y (2-9.7) | 6/88 (1 delayed union) | 18/24 |
| Ito et al., 2011 (45) | IV | 139 (84%) | 32 years (12–56) | Non-wb  Pwb+crutches  1 crutch | First 2w  At 2-4w  For 12w | - | - | HHS: 70🡪90 | - | 11y (5-20) | - | 18/24 |
| Yamanaka et al., 2011 (46) | IV | 1 (0%) | 20 years | Walk+crutches  Full weight | After 1w  After 8w | - | - | HHS: 85 🡪96 | Return to full activity (skiing) at 4m | 2y | - | 8/16 |
| Teratani et al., 2011 (47) | II | 42 (98%) | 54.6 years (50–65) | Active motion  Pwb+crutches  Full weight | POD2  POD3  At 8w | - | - | HHS: 69.6🡪 90.9; 71.1🡪 91.8 | - | 2y | - | 18/24 |
| Matheney et al., 2010 (48) | II | NA | NA | Pwb  Progressed to full weight | POD2/3  By 6-8w | - | - | - | - | 9y | 20/ 109 | 12/16 |
| Thawrani et al., 2010 (49) | IV | 76 (82%) | 15.6 years (11–21) | Pwb 9-13,6 kg  Full weight | For 6w  after | - | - | - | - | 2y | 3/76 major (osteonecrosis femoral head) | 12/16 |
| Troelsen et al., 2009 (50) | II | 96 (78%) | 29.9 years (14–57) | Pwb+crutches | First 8w | - | - | - | - | 6.8y | - | 12/16 |
| Keogh et al., 2008 (51) | NA | NA | NA | Toe-touch-wb | 6-8w | - | - | - | Return from 25w | - | - | n/a |
| Peters et al., 2006 (52) | IV | 73 (89%) | 25 years (13–45) | Pwb+crutches  Full+1 crutch  Walking w/o limp | For 6w  For 6w  At 12w | - | - | HHS: 54🡪87 | - | 46m | 10 major complications (nerve palsies) | 12/16 |
| Clohisy et al., 2005 (53) | IV | 97 (84%) | 28 years (13–47) | pwb | First 8w | - | - | HHS: 73.4🡪 91.3 | - | 4.2y | 2/13 (non-union; loss of fixation) | 12/16 |
| Ganz et al., 2004 (54) | NA | NA | NA | Mobi+ pwb 10kg+crutches  Walk with cane | POD3  After 8-10w | Soft splint | - | - | - | - | 14 major (1 non-union; 13 implant removal) | 10/16 |
| Hsieh et al., 2003 (55) | IV | 38 (79%) | 31 years (18–58) | Pwb+crutches  Walk with cane  w/o cane | POD4/5  After 6w  At 12w | - | - | Merle d’Aubigne and Postel hip score: 13.2🡪17 | - | 4.2y | 0 | 10/16 |
| Ko et al., 2002 (56) | IV | 36 (97%) | 29 years (14–53) | Mobi  Pwb  Full weight | POD4/5/6  At 1w  At 12-16w | - | - | mHHS: 59.1 +-15.8 🡪 87.97+-14.3 | - | 5.5y | - | 10/16 |
| Crowther et al., 2002 (57) | IV | 1 (100%) | 28 years | Touch-down-wb | 12w | Abduction brace 12w | - | - | - | 2y | - | 8/16 |
| Xiang et al., 2022 (58) | III | 67 (92%) | 32.4 years (18–52) | Active/passive exercise  Toe-touch-wb  Full weight | POD1  First 12w  after | - | - | - | - | 12m (12-36) | No major | 10/16 |
| Swarup et al., 2021 (59) | IV | 33 (97%) | 17 years (12–21) | 20% pwb  Wb as tolerated+crutches | For 4w  Until 6w | - | - | mHHS: 50🡪 88 | Return to all activities at 6m | Min. 1y | 0 | 10/16 |
| Matsuda et al., 2016 (60) | IV | 1 (0%) | 38 years | Discharged on  Pwb+crutches  Full weight | POD3  At 6w | - | - | - | - | - | 0 | 8/16 |
| Buchler et al., 2014 (61) | NA | NA | NA | Passive motion  Restricted wb 15kg  Increased wb | POD1  For 8w  after | - | - | - | - | - | - | n/a |
| Karashima et al., 2011 (62) | III | 163 (91%) | 36.5 years (20–57) | Pwb 10kg+crutches  Full weight | POD3  After 8w | - | - | HHS: 1. 73.9🡪 94.3; 2. 76.7🡪 94.7 | - | 70.9m; 70.6m | 12/ 191 (2 pubic non-union; 7 pubic fracture ; 1 ischial fracture) | 18/24 |
| Stetzelberger et al., 2021 (63) | III | 102 (72%) | 29 years (±11) | Pwb 15kg  Increase wb | For 8w  after | - | - | - | - | 22+-6y | - | 14/16 |
| Polkowski et al., 2014 (64) | IV | 134 (72%) | 30 years (18–60) | Mobi  Toe-touch-wb | POD2  POD3 | - | - | - | - | 26m (1-96) | - | 12/16 |
| Albers et al., 2013 (65) | III | 147 (75%) | 29 years (12–55) | Mobi+crutches  Pwb 15kg  Full weight | Early  For 8w  after | - | Rehab: 2-3m | Merle d’Aubigné: 15🡪16;  15🡪16 | - | 11y (10-14) | - | 20/24 |
| Mayman et al., 2002 (66) | IV | 8 (NA) | NA | Touchdown- wb+crutches  Progressive wb | For 6w  after | - | - | - | - | - | 0 | 10/16 |
| Salih et al., 2020 (67) | III | 200 (89%) | 28.8 years (13–48) | Pwb 20kg+crutches  Progressed to 30kg  Full weight+crutches  w/o crutches | For 6w  For 3w  After  By 12w | - | - | - | Impact exercise at 5-6m | 26m | 2.7% major ( 1 revision fixation; 2 stress fractures) | 10/16 |
| Leopold et al., 2023 (68) | III | 173 (87%) | 28 years (18–40) | Tip-touch-pwb  Increase to half body-weight  Increase to full weight | First 6w  Till 10^th^ w  10- 12w | - | - | Subjective hip value  41.9🡪77.9;  42.4🡪82.4 | - | 63m+-10 | 3/120 (implant migration) | 17/24 |
| Leopold et al., 2021 (8) | III | 84 (79%) | 27 years (15–45) | Tip-touch-pwb  Increased half body-weight  Increase to full weight | First 6w  7^th^-10^th^ w  10- 12w | - | - | - | - | 94d (70-112) | No major | 14/24 |

Table 1: Overview of postoperative weight-bearing protocols following periacetabular osteotomy (PAO)

This table summarizes the different studies evaluating weight-bearing protocols after PAO, including the duration of partial and full weight-bearing, the use of braces, total rehabilitation time, functional outcomes, participation in sports, follow-up duration, and major complications. Abbreviations: pwb – Partial weight-bearing; wb – Weight-bearing; POD – Postoperative day; HHS – Harris Hip Score; mHHS – Modified Harris Hip Score
