# Supplementary figures and images for: Postoperative weight-bearing restrictions and rehabilitation after periacetabular osteotomy: a systematic review
Source: J Orthop Surg Res. 2025 Oct 29;20:944. doi: 10.1186/s13018-025-06448-x (PMC12573821; doi:10.1186/s13018-025-06448-x)

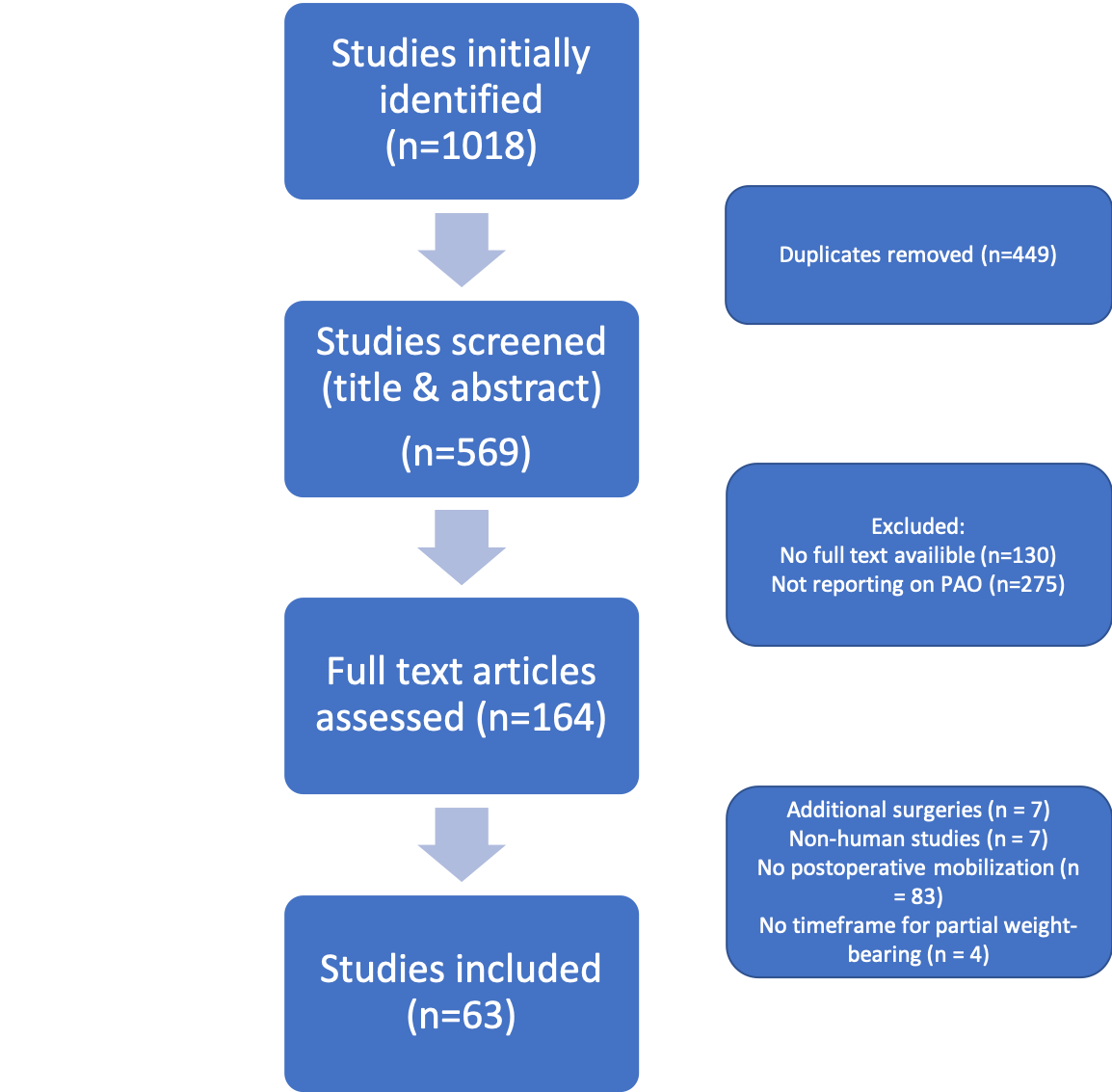

Supplement: Supplementary file 2 — Supplementary Material 2 [file 13018_2025_6448_MOESM2_ESM.docx]
